# Can the Xpert MRSA/SA BC assay be used as an antimicrobial stewardship tool? A prospective assay validation and descriptive impact assessment study in a South African setting

**DOI:** 10.1186/s12879-021-05857-7

**Published:** 2021-02-15

**Authors:** Kessendri Reddy, Andrew Whitelaw

**Affiliations:** Division of Medical Microbiology and Immunology, Department of Pathology, Faculty of Medicine and Health Sciences, Stellenbosch University/National Health Laboratory Services Tygerberg, Cape Town, South Africa

**Keywords:** *Staphylococcus aureus*, Xpert MRSA/SA BC, Staphylococcal bloodstream infection, Antimicrobial stewardship

## Abstract

**Background:**

Positive blood cultures showing Gram positive cocci in clusters signifies either *Staphylococcus aureus* or the less-virulent coagulase-negative staphylococci. Rapid identification and methicillin susceptibility determination with the Xpert MRSA/SA BC assay can improve management of *S. aureus* bloodstream infection and reduce inappropriate antibiotic use.

**Methods:**

We prospectively evaluated the Xpert MRSA/SA BC assay in comparison with culture, on samples referred to our laboratory in the Western Cape, South Africa. We interviewed attending clinicians upon culture result availability, to assess antibiotic choices and estimate potential impact of the assay.

**Results:**

Of the 231 samples included, there was 100% concordance between the Xpert MRSA/SA BC assay and culture (methicillin-resistant *S. aureus* 15/15, methicillin-susceptible *S. aureus* 42/42, coagulase-negative staphylococci 170/170). Time to final result could be reduced by approximately 30 h with the assay. Of the 178 patients with adequate antibiotic history, optimisation of antistaphylococcal therapy could have occurred more than 1 day sooner in 68.9% with *S. aureus* bloodstream infection (31/45, 95% CI 53.2–81.4%). Six of the 11 patients with methicillin-resistant *S. aureus* bloodstream infection (54.5%) could have received anti-MRSA cover sooner. Fifty-four days of antibiotic therapy could have been spared, equating to 0.3 days (95% CI, 0.2–0.4) saved per patient, driven by broad-spectrum beta-lactams (32 days, in 18.0% of the cohort).

**Conclusion:**

This assay has potential as an antimicrobial stewardship tool; costing and impact on clinical outcome in patients with *S. aureus* bloodstream infection should be assessed.

**Supplementary Information:**

The online version contains supplementary material available at 10.1186/s12879-021-05857-7.

## Background

*Staphylococcus aureus* is a common cause of bloodstream infection (BSI) [1], with a mortality rate of 20–40% [1]. In contrast, coagulase-negative staphylococci (CoNS) are generally regarded as skin commensals of low pathogenic potential, although they can be clinically significant in selected circumstances.

Differentiating between CoNS and *S. aureus* in positive blood culture broths showing Gram positive cocci in clusters (GPCC) on Gram stain, typically requires assessment of morphological and biochemical characteristics after overnight incubation. In addition, susceptibility testing is required to differentiate between methicillin-susceptible and methicillin-resistant *S. aureus* (MSSA and MRSA, respectively). These requirements can result in delayed initiation of appropriate therapy in patients with *S. aureus* BSI, with potentially adverse outcomes including secondary infectious complications [2], a higher mortality rate and prolonged hospital stay [1]. Conversely, inappropriate antibiotic administration unnecessarily exposes the patient to potential adverse effects of the medication, alters gastrointestinal flora, and also has ecological effects, including contributing to antibiotic selective pressure which drives resistance.

The Xpert MRSA/SA BC System (Cepheid, Sunnyvale, California) differentiates between MRSA, MSSA and CoNS from positive blood cultures within approximately 1 h, using a real-time, semi-automated, nucleic acid-based test which targets *spa* and the SCC*mec-orfX* junction (for *S. aureus*), and *mecA* (for methicillin resistance). Published studies, summarised in one paper [3], report sensitivities and specificities of 96.4–100% and 98.0–100% respectively, for the detection of MSSA, and values of 87.5–100% and 98.3–100% respectively, for detection of MRSA. Initial concerns regarding undercalling of MRSA with specific SCC*mec* variants, were resolved in 2013 [3].

We evaluated the Xpert MRSA/SA BC System (Xpert) and assessed the potential impact of implementation of this test as an antimicrobial stewardship tool in the patient population served by our laboratory in Cape Town, South Africa.

## Methods

### Study aims, design and setting

We aimed to assess the performance and role of the Xpert in facilitating more appropriate antibiotic use in patients with *S. aureus* bloodstream infection, and reducing inappropriate antibiotic therapy in those with *S. aureus* and with coagulase-negative staphylococci. We performed a prospective observational study to evaluate the Xpert against the reference method of culture-based techniques, and to describe the potential impact of implementation of this assay in our setting by combining clinical history with attending clinician survey.

The study took place from January to June 2016 at the National Health Laboratory Service (NHLS) Microbiology laboratory at Tygerberg Hospital in the Western Cape, South Africa. Tygerberg Hospital is a 1384-bedded referral hospital serving the northern and eastern subdistricts of the Cape Metro region, and the surrounding rural districts, including 4 regional hospitals and 17 district hospitals.

Blood culture bottles were submitted from facilities throughout this drainage area at the clinicians’ discretion. The automated blood culture system in use is the BacT/Alert 3D Microbial Detection System (bioMérieux, Marcy L’Étoile, France) which includes anaerobic (FN Plus), aerobic (FA Plus) and paediatric bottles (PF Plus). These were kept at room temperature during transport, and were incubated as soon as possible after specimen receipt. After flagging positive, Gram stains are performed on an aliquot of the broth. All patients with positive blood cultures exclusively showing GPCC on Gram stain, were included. Blood culture specimens showing mixed morphology on Gram stain, and duplicate blood culture samples from the same patient, were excluded.

Paediatric patients were defined as patients below the age of 13 years.

### Classification and management of suspected sepsis

Patients with positive blood cultures (indicating suspected sepsis) were defined as having community-acquired (CA) infection if the culture was collected < 72 h after admission, and hospital acquired (HA) infection thereafter; admission within the preceding 6 weeks was also considered a criterion for HA infection [4] .

Empiric management of CA infections was based on local guidelines, with ceftriaxone or amoxicillin-clavulanate used for most infections; cloxacillin was recommended where *S. aureus* was likely. During the study period, standard-of-care was to administer a carbapenem +/− vancomycin (for suspected MRSA) for presumed hospital-acquired sepsis.

Regional hospital guidelines in our drainage area advise a targeted approach of a semi-synthetic beta-lactamase stable penicillin (cloxacillin) for MSSA BSI, with vancomycin advised for MRSA BSI.

### Validation

#### Laboratory processing

Consecutive samples were processed in parallel using culture-based methods and Xpert, during weekdays between 8 am and 4 pm. The investigator performing the Xpert was blinded to the culture results.

Culture-based methods entailed inoculation and overnight incubation of basic enriched agar media; Kirby-Bauer disk diffusion testing of an aliquot of broth against a standard anti-staphylococcal antibiotic panel; and identification using DNAse and Mannitol Salt Agar (MSA) plates. A rapid latex agglutination test was performed using the Pastorex Staph Plus kit (Bio-Rad, Hercules, California) for indeterminate identification results. VITEK 2 Gram positive ID confirmation (bioMérieux, Marcy L’Étoile, France) was used at the discretion of the clinical microbiologist, and methicillin susceptibility was determined by cefoxitin disk diffusion testing using the Clinical and Laboratory Standards Institute (CLSI) guidelines [5].

Batched Xpert testing was performed on blood culture bottles, which were stored at 35 °C following culture-based processing. The Xpert MRSA/SA BC G3 version 5 (Cepheid, Sunnyvale, California) was performed as per manufacturer’s instructions. Briefly, 50 μl of blood culture broth was added to the vial containing the elution reagent and vortexed for 10 s. The contents of the vial were transferred to the test cartridge and loaded into the module. Xpert testing was performed as soon as possible after positivity; there was a maximum delay of 80 h for bottles flagging positive over a weekend. The results of the Xpert assay were not made available to the clinicians.

The results of these methods were interpreted as summarised in Table 1.

**Table 1:** Laboratory processing result interpretation of positive blood cultures containing Gram positive cocci in clusters

	Culture-based methods		Xpert MRSA/SA BC	
	Species identification	Methicillin susceptibility determination	Species identification	Methicillin susceptibility determination
Methicillin-resistant *Staphylococcus aureus*	DNAse and MSA positive; OR DNAse and MSA discrepant^a^ AND *S. aureus* on VITEK 2	Cefoxitin resistant on disk diffusion testing^b^	Detection of *spa* gene	Detection of *mecA* and the SCC*mec-orfX* region
Methicillin-susceptible *Staphylococcus aureus*	DNAse and MSA positive; OR DNAse and MSA discrepant^a^ AND *S. aureus* on VITEK 2	Cefoxitin susceptible on disk diffusion testing^b^	Detection of *spa* gene	Absence of *mecA*
Coagulase-negative staphylococci	DNAse and MSA negative; OR DNAse and MSA discrepant AND no agglutination using Pastorex Staph Plus; OR CoNS species on VITEK 2	Cefoxitin disk diffusion testing result^b^	Absence of *spa* gene	Presence (resistant) or absence (susceptible) of *mecA*

*DNAse* Deoxyribonuclease, *MSA* Mannitol Salt Agar, *CoNS* Coagulase-negative staphylococcus

^a^Pastorex Staph Plus-tested isolates exhibiting agglutination were all subjected to VITEK 2 confirmation. Isolates with no agglutination were considered coagulase-negative staphylococci, or were subjected to VITEK 2 identification at the discretion of the clinical microbiologist

^b^Cefoxitin category determined by clinical breakpoints outlined in the Clinical and Laboratory Standards Institute M100 document (2016)

### Impact assessment and definitions

As per standard practice, clinicians were contacted with the Gram stain result if the available clinical information or previous results suggested *S. aureus* sepsis, or if the patient was admitted in an Intensive Care Unit (ICU). In all other cases, clinicians were contacted with the culture result, whether *S. aureus* or CoNS.

Basic clinical and demographic information were obtained, including an antibiotic history. We surveyed the attending clinicians to assess whether knowledge of the culture and susceptibility result on the day the bottle flagged positive would have impacted antibiotic choice. This was categorised as:

- *Modification:* a change from an ineffective to a more effective agent, or the addition of a semisynthetic penicillin (MSSA) or glycopeptide (MRSA);
- *De-escalation:* a change in antibiotic to a narrower-spectrum, targeted antistaphylococcal agent, or cessation of some or all antibiotics;
- *No change:* no change to the empiric antibiotic regimen, or no antibiotics prescribed if the patient was not receiving antibiotics.

We regarded cephalosporins (with the exception of ceftazidime), beta-lactam–beta-lactamase inhibitors, carbapenems and vancomycin as being active against MSSA. Penicillin, ampicillin, amoxicillin, and ceftazidime were the beta-lactam agents regarded as being ineffective against MSSA.

CoNS were considered significant only if reported as the likely cause for the patient’s clinical condition, as judged by the attending clinician.

Days of antibiotic therapy saved were also evaluated during clinician interview, based on the empiric antibiotic regimen commenced and on clinician opinion as to whether an earlier result would have impacted antibiotic choice or duration (i.e. if the Xpert had been utilised as soon as the bottle flagged positive, leading to a “final” result being available shortly after Gram stain availability).

### Statistical analysis

Microsoft Excel, VassarStats (www.vassarstats.net) and EpiCalc 2000 v1.02 (Brixton Books, South London, UK) were used, with an α-level of 0.05 chosen. Continuous numerical variables were summarised using means and standard deviations for normally distributed variables, or medians and interquartile ranges (IQR) for variables not following a normal distribution. Binary categorical variables were summarised using proportions and 95% confidence intervals (CI). Comparison of selected independent proportions were evaluated using the z-test.

## Results

Our laboratory processed 2822 positive blood culture bottles between January and June 2016. Of these, 1158 (41.0, 95% CI 39.2–42.9%) demonstrated only GPCC on Gram stain and 231 samples were included in the study (19.9, 95% CI 17.7–22.4%) due to convenience sampling. Selected variables were compared between the participants included in the study and all patients for whom blood cultures were submitted showing Gram positive cocci in clusters on Gram stain, between January and June 2016. No differences in age, gender or ward location per organism type at blood culture sampling were noted, with the exception of a larger proportion of coagulase-negative staphylococci from patients admitted to surgical wards (15.9% vs 9.2%, p 0.006) and high care units/ICU (15.9% vs 8.3%, p 0.002) in the study population. Contribution of the bottle types is outlined in Table 2.

**Table 2:** BacT/Alert blood culture bottles included in the diagnostic evaluation of Xpert MRSA/SA BC

Bottle Type	n (%)	95% confidence interval
FA Plus	132 (57.1)	50.5–63.6
FN Plus	19 (8.2)	5.2–12.7
PF Plus	80 (34.6)	28.6–41.2
Total	231	–

*Xpert* Xpert MRSA/SA BC assay, *FA Plus* FAN Aerobic Plus, *FN Plus* FAN Anaerobic Plus, *PF Plus* Paediatric FAN Plus

### Validation

A valid Xpert result was initially obtained from 225/231 bottles (97.4, 95% CI 94.5–98.8%). Of the remaining 6 assays, 2 (2.6, 95% CI 1.2–5.6%) were invalid (failure of internal control) and were not repeated. Four were resulted as “error” with signal loss after initial amplification; two provided a valid result after repeat testing. The conservative failure rate was 1.7% (95% CI 0.7–4.4%), with 227 bottles yielding an interpretable result.

MSSA was present in 42 samples (18.5, 95% CI 14.0–24.1%) and MRSA in 15 (6.6, 95% CI 4.1–10.6%). CoNS were present as the sole isolate in 169 patients, and were part of mixed cultures in an additional 4 cases. Three of these 4 mixed cultures contained MSSA together with CoNS, were detected as MSSA by the Xpert, and were included as MSSA in the analysis. The fourth culture contained *Klebsiella pneumoniae* (Gram negative bacilli missed on Gram stain) and a CoNS; the Xpert correctly assessed the CoNS.

There was 100% concordance between the systems for the identification of MSSA, MRSA and CoNS. The Xpert assay had an overall sensitivity of 100% (57/57; 95% CI 93.7–100%), and specificity of 100% (170/170, 95% CI 97.8–100%). The positive and negative predictive values of the assay were both 100% (95% CIs 93.7–100% and 97.8–100% respectively). Analysis by MSSA, MRSA and CoNS is summarised in Supplementary Table 1.

We performed a subanalysis of *mecA* and methicillin resistance in CoNS, for the 169 isolates in which cefoxitin susceptibility on culture was known. The gene target, *mecA,* was detected in 85 of 88 CoNS isolates that were cefoxitin-resistant on culture (96.6%; 95% CI 90.5–98.8%) giving a positive predictive value of 86.7% (95% CI 78.6–92.1%). The specificity of detection of *mecA* in CoNS on Xpert was 84.0% (95% CI 74.5–90.4%) with a negative predictive value of 95.8% (95% CI 88.3–98.6%).

### Potential impact of implementation

The median time between blood culture positivity and final result authorisation was 31.3 h (interquartile range (IQR) 20.7–42.5 h).

Of the 227 patients included, 50.7% (115/227, 95% CI 44.0–57.3%) were males. The median age of the adults included was 43.0 years (IQR 31.0–58.0); the median age of the paediatric population was 71.5 days (IQR 17.8–325.0). Twenty-five adults (25/151, 16.6, 95% CI 11.2–23.7%) and 10 children (10/76, 13.2, 95% CI 6.8–23.3%) were admitted to an ICU at the time of blood culture collection. Sepsis was CA in 51.1% (116/227, 95% CI 44.4–57.8%), HA in 38.3% (87/227, 95% CI 32.0–45.0%); and unknown in the remaining 10.6% (24/227, 95% CI 7.0–15.5%). Only 1/11 with MRSA BSI reportedly had CA onset of infection (9.1, 95% CI 0.5–42.9%).

Adequate history regarding source of sepsis was obtained for 195 patients. Sixty-two patients (31.8, 95% CI 25.4–38.9%) had an unclear source of sepsis. Respiratory tract infection was the most commonly identified suspected source overall (56/195, 28.7, 95% CI 22.6–35.7%) and in CA-infection (33/101, 32.7, 95% CI 23.9–42.8%). For HA-infection, 29 patients (36.3, 95% CI 26.0–47.8%) had no clear source of sepsis; of those with known primary sources, the major foci were the respiratory tract and skin or skin structures (both 18/80 patients, 22.5, 95% CI 14.2–33.5%).

Information on the suspected source of sepsis was obtained in 46 patients with *S. aureus* BSI. The most commonly identified suspected source of sepsis (in isolation or as part of two potential sources) was skin or skin structure infection (*n* = 16, 34.8%), followed by suspected respiratory tract infection (*n* = 11, 23.9%). Source was undetermined at the time of clinician interview in 8 patients with *S. aureus* BSI (17.4%).

Characteristics of the included patients can be found in Supplementary Table 2.

#### Impact on antibiotic administration

**Fig. 1:**
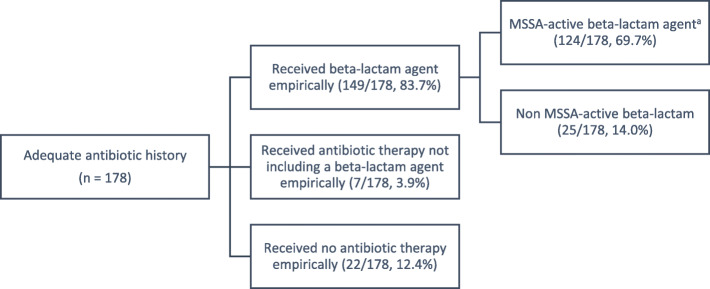
Empiric antibiotics in patients with GPCC on blood culture and adequate antibiotic history. MSSA: methicillin-sensitive *Staphylococcus aureus;* GPCC: Gram positive cocci in clusters. ^a^Ceftriaxone/cefotaxime were included as MSSA-active beta-lactam agents when used empirically

Adequate antibiotic history was obtained in 181 patients, including 34 with MSSA and 11 with MRSA BSI. Three patients were unclassified due to discordance between culture result, clinical history and antibiotic therapy; these were excluded from the analysis (additional notes in Supplementary Table 3). Empiric antibiotic therapy choices for the remaining 178 patients (including one with significant CoNS BSI resulting from a central line infection) are outlined in Fig. 1.

**Fig. 2:**
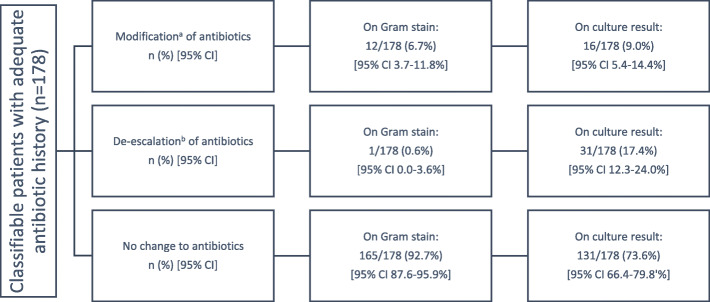
Antibiotic progression at Gram stain availability and final culture result availability. CI: confidence interval. ^a^ Modification: change from an ineffective to a more effective agent, or the addition of a semisynthetic penicillin (methicillin-susceptible *S. aureus*) or glycopeptide (methicillin-resistant *S. aureus*); ^b^ De-escalation: change to a narrower-spectrum, targeted antistaphylococcal agent, or cessation of some or all antibiotics

The antibiotic changes over time are summarised in Fig. 2 (whole cohort) and Table 3 (subgroup with *S. aureus* BSI). The impact of the Xpert could be discerned by proxy if culture result availability approximated availability of the Gram stain result (i.e. final result available about an hour after Gram stain availability, in place of the overnight incubation required by reference methods). Analysis by organism category (MRSA, MSSA or CoNS) can be found in Supplementary Table 4.

**Table 3:** Antibiotic progression in patients with Staphylococcus aureus bloodstream infection with antibiotic history (*n* = 45)

	Methicillin-sensitive *S. aureus* (*n* = 34)			Methicillin-resistant *S. aureus* (n = 11)		
	Empiric Choice n (%) [95% CI]	In response to Gram result n (%) [95% CI]	On culture result n (%) [95% CI]	Empiric choice n (%) [95% CI]	In response to Gram result n (%) [95% CI]	On culture result n (%) [95% CI]
Patients receiving an agent with activity against the organism isolated^a^	27 (79.4) [61.6–90.7]	29 (85.3) [68.2–94.5]	34 (100) [87.4–100]	3 (27.3) [7.3–60.7%]	5 (45.5) [18.1–75.4]	11 (100.0) [67.9–100.0]

*MRSA* Methicillin-resistant *Staphylococcus aureus*, *MSSA* Methicillin-sensitive *Staphylococcus aureus, CI* Confidence interval

^a^Empiric cover for MSSA included any agent with anti-MSSA activity, including ceftriaxone/cefotaxime for MSSA. Empiric cover for MRSA consisted of vancomycin in our setting

#### MRSA

Most patients with MRSA BSI did not receive an MRSA-active agent until release of the final culture result (Table 3). Cessation of an additional antimicrobial agent occurred in 7 patients on final culture result (63.6, 95% CI 31.6–87.6%); this could have occurred at least 1 day sooner with Xpert.

#### MSSA

For the 34 patients who had MSSA BSI, notable aspects of the antibiotic regimen include:

- Empiric cover: Empiric vancomycin was prescribed unnecessarily in 3/34 (8.8, 95% CI 2.3–24.8%); this could have been avoided if Xpert was used.
- In response to Gram stain availability: 2/34 (5.9, 95% CI 1.0–21.1) received modifications to their antibiotic regimens in response to Gram stain result availability. In one case, vancomycin was started when the Gram stain became available, for suspected HA-sepsis. In the second, a non MSSA-active beta-lactam agent (ampicillin) was changed to an MSSA-active agent (cefotaxime).
- In response to final culture result availability: Overall, there was a change in antibiotic therapy on final result in 25 patients (73.5, 95% CI 55.4–86.5%). These could have occurred at least 1 day earlier had the Xpert assay been used.
  - ○ De-escalation to a semisynthetic penicillin in 18/25 (72.0, 95% CI 50.4–87.1%)
  - ○ Modification to a more effective agent in the remaining 7/25 (28.0, 95% CI 12.9–49.6%)

#### CoNS

Antibiotics were stopped in 13/133 patients on release of the final result indicating CoNS (9.8, 95% CI 5.5–16.5%).

#### Antimicrobial stewardship impact

Overall, 31/45 patients with *S. aureus* BSI and known antibiotic history could have received more appropriate antistaphylococcal therapy 1 day sooner, with adoption of the Xpert assay (68.9, 95% CI 53.2–81.4%). Eleven of the 45 patients (24.4, 95% CI 13.4–39.9%) were not receiving an agent with activity against their pathogen following Gram stain result availability (6 patients with MRSA and 5 with MSSA BSI).

#### Days of therapy potentially saved

In the 178 patients with known antibiotic history, at least 32 days of exposure to a broad spectrum beta-lactam (beta-lactam-beta-lactamase inhibitor combination, extended-spectrum cephalosporin or carbapenem) could have been spared with earlier knowledge of the final result, equating to a reduction in broad spectrum beta-lactam use in 18.0% of the patients in this study (32/178, 95% CI 12.8–24.6%).

A further 5 days of vancomycin use could have been avoided, and 17 days of antibiotic therapy could have been spared for the other agents (Supplementary Table 5). This equates to a reduction of 0.3 antibiotic days per patient (95% CI, 0.2–0.4).

In total, at least 54 days of antibiotic therapy could have been spared in the 178 patients with GPCC on blood culture in this study.

## Discussion

### Evaluation

The Xpert MRSA/SA BC system reliably differentiated *S. aureus* from CoNS. The assay showed a high sensitivity and specificity, was easy to use and is potentially implementable in a South African setting, where Xpert modules are widely available as part of the national programme for diagnosis of tuberculosis.

Methicillin resistance was detected with 100% accuracy in *S. aureus*; the small MRSA sample size precludes firm conclusions. Methicillin resistance in *S. aureus* is chiefly mediated by *mecA* at present, although *mecC-*mediated resistance has been reported in 0.004% of human isolates [6]. The assay also performed well for MSSA BSI, showing 100% concordance with culture-based testing. The error rate of 1.7% is similar to previous reports [3, 7] and is acceptable. Although this assay has not been rigorously tested for the detection of methicillin resistance in CoNS to date, the negative predictive value of 95.8% in this context may be useful in selected cases of potentially significant CoNS bloodstream infection.

We recommend conventional culture to be performed at least limitedly in parallel, for mixed cultures, as a backup in case of an unsuccessful Xpert result and for further antimicrobial susceptibility testing or surveillance. Genotype-phenotype mismatch, with the Xpert assay incorrectly reporting a susceptible methicillin result due to insertions in the SCC*mec-orfX* junction region, has been reported in 3 *S. aureus* isolates from the United States [8], and misclassification of *S. aureus* as CoNS in 2 isolates from Australia [9]. Studies investigating the prevalence of mutations that may affect the targets of this assay in a South African setting are needed to define the role of this assay more accurately.

### Potential impact of implementation

To our knowledge, this is the largest study assessing the potential impact of this assay to date. The median time saving to final result with use of this assay was approximately 30 h if the Xpert assay was performed immediately after microscopy, in line with previous estimates of a time saving of 24–48 h [2, 10]. This crude estimate can be influenced by factors such as workflow. Antibiotic therapy was optimised on availability of final result in 68.9% with *S. aureus* BSI. This impact was most clearly demonstrated in patients with MRSA BSI, where more than 50% received anti-MRSA therapy only in response to the final culture result. A more modest impact could be seen for patients with MSSA BSI (approximately 21%); however, it must be noted that this is a conservative estimate as all beta-lactam agents were considered to have activity against MSSA in this analysis, although they may not be equally suitable for the treatment of MSSA BSI and some are associated with a higher odds of death [11].

More rapid administration of appropriate therapy can significantly impact mortality. In one study, the mean mortality rate was 59% for patients with MRSA BSI who were initially started on a semisynthetic penicillin, as opposed to 23–36% for patients initially started on vancomycin [12]. The mean mortality rate was as low as 12% in patients empirically placed on a semisynthetic penicillin, who cultured MSSA [12]. Future studies should specifically assess the clinical impact of Xpert on more appropriate *S. aureus* therapy.

We estimated a reduction in broad spectrum beta-lactam antibiotic use in 18.0% of the cohort where antibiotic history was available, with 54 days of antibiotic therapy saved. This translates to a modest reduction in antibiotic days of therapy of 0.3 days per patient. When combined with the benefits shown in optimising therapy for *S. aureus* BSI infection, this may still be regarded as advantageous, particularly when considered with the consequences of unnecessary antibiotic prescription.

We initially expected this assay to be of most use in reducing antibiotic therapy in patients with CoNS, as has been reported previously [13], but this finding was not replicated. The vast majority of CoNS bloodstream isolates were regarded as being not clinically significant in this cohort, in keeping with the high rate of blood culture contamination in our setting [14]. Clinicians thus disregarded this “contaminated” result and continued the empiric antibiotic regimen that had been commenced based on the initial clinical assessment. Decisions regarding antibiotics are also governed by other factors not measured in this study, such as the clinical response to antibiotic therapy and additional test results. Although availability of relevant rapid diagnostic tests such as the Xpert is a core element to antimicrobial stewardship programmes in healthcare facilities in low-middle income countries [15], good quality microbiological sampling is also crucial and results in more appropriate use of laboratory resources. Ongoing educational interventions to optimise blood culture sampling (and reduce blood culture contamination) is a relatively cost-effective and easily implementable measure that can be instituted before turning to more costly solutions, and it is important that this remains a focus of antimicrobial stewardship programmes in resource-limited settings.

The potential benefits described must be weighed against the expense of the assay, including additional labour, infrastructure and consumable costs. The total cost of the test may be prohibitive in a resource-constrained setting such as our own.

Limitations to this study include that the *S. aureus* BSI rate was only 25.1% of all blood cultures with Gram positive cocci in clusters, and that methicillin resistance was detected in 26.3% of all *S. aureus*-containing blood cultures (similar to a contemporaneous study in our setting (27.1%) [16]); this resulted in modest absolute numbers of MSSA and MRSA BSI. This may confer a more moderate impact of test performance than would be seen in higher prevalence settings. CA-MRSA is also not common in our setting currently [17]. Secondly, complete clinical records were not available for all patients, prohibiting case-by-case assessment of whether antibiotic changes occurred as a result of the microbiological results or the evolving clinical picture. Furthermore, clinician recall bias may have influenced these results. Thirdly, other patient comorbidities, such as renal failure and severity of illness, may have contributed to the choice of agents used and the decision to modify antibiotic therapy; this could not be easily assessed. Fourthly, we sampled a proportion of those eligible for inclusion; although we compared these populations for all the key variables that would be available in a routine diagnostic laboratory setting (thereby limiting selection bias), other variables, such as source of sepsis, were not taken into account. Additionally, significant CoNS BSI may have been underestimated in this study, as clinicians in our setting infrequently adhere to recommendations for multiple blood cultures to be submitted in the investigation of sepsis. Finally, most beta-lactam agents were considered to have anti-MSSA activity for the purposes of this study, which may result in an underestimate of the clinical benefit of the assay for MSSA BSI.

## Conclusion

The Xpert MRSA/SA BC assay performed well in differentiating *S. aureus* from CoNS on positive blood cultures with Gram positive cocci in clusters and in detecting methicillin resistance in *S. aureus,* and the assay may be an effective antimicrobial stewardship tool. Further studies may need to be performed in areas with differing blood culture contamination and MRSA rates. We demonstrated potential benefit in reducing time to appropriate therapy in the majority of patients with *S. aureus* bloodstream infection. Antibiotic modification based on the clinical response may have played a role in amplifying or attenuating the benefit observed in this study. We showed a modest reduction in antibiotics prescribed; studies limited to a more homogenous population may show a greater benefit. Additional studies assessing potential impact should focus on individual patient clinical status and outcomes, and should take into account source control, cost reduction in terms of antibiotic administration and hospital stay, and comprehensive costing.

## Supplementary Information

**Additional file 1: Table S1.** Diagnostic accuracy of the Xpert MRSA/SA BC assay on positive blood cultures containing Gram positive cocci in clusters on Gram stain, compared with culture-based methods (*n* = 227). A summary of the results of the evaluation of the Xpert assay for methicillin-resistant *S. aureus,* methicillin-susceptible *S. aureus* and coagulase-negative staphylococci.

**Additional file 2: Table S2.** Characteristics of the included patients with blood cultures containing Gram positive cocci in clusters (*n* = 227). A summary of the basic demographic characteristics of the patients included in the diagnostic evaluation of the Xpert MRSA/SA BC assay, and the suspected source of sepsis in the 195 patients with clinical history on source of sepsis.

**Additional file 3: Table S3.** Patients with Gram positive cocci in clusters on blood culture and adequate antibiotic history who were excluded from impact analysis due to discordance between culture result, clinical history and antibiotic therapy (*n* = 3). Further details on the three patients with adequate clinical history pertaining to antibiotic use who were excluded from analysis due to inconsistency between culture result, clinical history and antibiotic therapy.

**Additional file 4: Table S4.** Evolution of antimicrobial therapy on Gram stain and culture result availability, in patients with Gram positive cocci in clusters on blood culture and known antibiotic history (*n* = 178). Action (modification/de-escalation/no change) in the 178 patients with known antibiotic history, in response to Gram stain and culture result release.

**Additional file 5: Table S5.** Contribution of antibiotic agents to the days of antibiotic therapy saved in patients with known antibiotic history (*n* = 178). A summary of antibiotic days potentially saved, by antibiotic type, in this cohort with use of the Xpert MRSA/SA BC assay.

## Data Availability

The anonymised datasets used and/or analysed during the current study are available from the corresponding author on reasonable request.

## Abbreviations

BSI: Bloodstream infection
CA: Community-acquired
CI: Confidence interval
CoNS: Coagulase-negative staphylococci
GPCC: Gram positive cocci in clusters
HA: Hospital-acquired
ICU: Intensive care unit
MRSA: Methicillin-resistant *Staphylococcus aureus*
MSSA: Methicillin-susceptible *Staphylococcus aureus*
Xpert: Xpert MRSA/SA BC
