# Supplementary material for: Can the Xpert MRSA/SA BC assay be used as an antimicrobial stewardship tool? A prospective assay validation and descriptive impact assessment study in a South African setting
Source: BMC Infect Dis. 2021 Feb 15;21:177. doi: 10.1186/s12879-021-05857-7 (PMC7885373; doi:10.1186/s12879-021-05857-7)
Supplement: Supplementary file 1 — Additional file 1: Table S1. Diagnostic accuracy of the Xpert MRSA/SA BC assay on positive blood cultures containing Gram positive cocci in clusters on Gram stain, compared with culture-based methods (n = 227). A summary of the results of the evaluation of the Xpert assay for methicillin-resistant S. aureus, methicillin-susceptible S. aureus and coagulase-negative staphylococci. [file 12879_2021_5857_MOESM1_ESM.docx]

**Additional file 1 (Supplementary material):**

Can the Xpert MRSA/SA BC assay be used as an antimicrobial stewardship tool? A prospective assay validation and descriptive impact assessment study in a South African setting

*Supplementary Table 1: Diagnostic accuracy of the Xpert MRSA/SA BC assay on positive blood cultures containing Gram positive cocci in clusters on Gram stain, compared with culture-based methods (n=227)*

|  | Sensitivity  *n (%; 95% CI*) | Specificity  *n (%, 95% CI*) | PPV  n *(%, 95% CI*) | NPV  *n (%, 95% CI*) |
| --- | --- | --- | --- | --- |
| *Staphylococcus aureus* | 57/57  *(100; 93.7-100)* | 170/170  *(100; 97.8-100)* | 57/57  *(100, 93.7-100)* | 170/170  *(100; 97.8-100)* |
| MSSA | 42/42  *(100, 91.6-100)* | 185/185  *(100, 98.0-100)* | 42/42  *(100, 91.6-100)* | 185/185  *(100, 98.0-100)* |
| MRSA | 15/15  *(100, 79.6-100)* | 212/212  *(100, 98.2-100)* | 15/15  *(100, 79.6-100)* | 212/212  *(100, 98.2-100)* |
| CoNS | 170/170  *(100, 97.8-100)* | 57/57  *(100, 93.7-100)* | 170/170  *(100, 97.8-100)* | 57/57  *(100, 93.7-100)* |

Data are presented as n (%)

CI: confidence interval; PPV: positive predictive value; NPV: negative predictive value; MRSA: methicillin-resistant *S. aureus*; MSSA: methicillin-sensitive *S. aureus*; CoNS: coagulase-negative staphylococci
