# Supplementary material for: Can the Xpert MRSA/SA BC assay be used as an antimicrobial stewardship tool? A prospective assay validation and descriptive impact assessment study in a South African setting
Source: BMC Infect Dis. 2021 Feb 15;21:177. doi: 10.1186/s12879-021-05857-7 (PMC7885373; doi:10.1186/s12879-021-05857-7)
Supplement: Supplementary file 2 — Additional file 2: Table S2. Characteristics of the included patients with blood cultures containing Gram positive cocci in clusters (n = 227). A summary of the basic demographic characteristics of the patients included in the diagnostic evaluation of the Xpert MRSA/SA BC assay, and the suspected source of sepsis in the 195 patients with clinical history on source of sepsis. [file 12879_2021_5857_MOESM2_ESM.docx]

**Additional file 2 (Supplementary material):**

Can the Xpert MRSA/SA BC assay be used as an antimicrobial stewardship tool? A prospective assay validation and descriptive impact assessment study in a South African setting

*Supplementary Table 2: Characteristics of the included patients with blood cultures containing Gram positive cocci in clusters (n=227)*

| Characteristic | *n (%) [95% confidence interval]* | |
| --- | --- | --- |
| Age/Ward |  | |
| *Adult* | 151 (66.5) [60.0-72.6] | |
| ICU, high care and burns unit ICU ^a^ | 25 (16.6) [11.2-23.7] | |
| *Paediatric* | 76 (33.5) [27.5-40.1] | |
| ICU, high care^b^ | 10 (13.2) [6.8-23.3] | |
| Gender |  | |
| Male | 115 (50.7) [44.0-57.3] | |
| Type of sepsis |  | |
| Community-acquired | 116 (51.1) [44.4-57.8] | |
| Hospital-acquired | 87 (38.3) [32.0-45.0] | |
| Not known | 24 (10.6) [7.0-15.5] | |
| Presumed source of sepsis (n=195)^c^ |  | |
| Respiratory tract | 56 (28.7) [22.6-35.7] | |
| Skin and soft tissue | 19 (9.7) [6.1-15.0] | |
| Abdominal/Gastrointestinal tract | 19 (9.7) [6.1-15.0] | |
| Urinary tract | 12 (6.2) [3.4-10.8] | |
| Line-associated | 12 (6.2) [3.4-10.8] | |
| Bone/joint | 7 (3.6) [1.6-7.6] | |
| Head/neck | 6 (3.1) [1.3-6.9] | |
| Cardiovascular (including infective endocarditis) | 4 (2.1) [0.7-5.5] | |
| Trauma-related | 3 (1.5) [0.4-4.8] | |
| Unknown | 62 (31.8) [25.4-38.9] | |
| Presumed source of sepsis for those with *Staphylococcus aureus* bloodstream infection (n=46)^d^ | | |
| Skin or skin structure | | 16 (34.8) [21.8-50.3] |
| Respiratory tract | | 11 (23.9) [13.1-39.1] |
| Bone/joint | | 7 (15.2) [6.8-29.5] |
| Infective endocarditis | | 3 (6.5) [1.7-18.9] |
| Other^e^ | | 5 (10.9) [4.1-24.4] |
| Unknown | | 8 (17.4) [8.3-32.0] |

ICU: Intensive Care Unit

^a^Percentage of total adult patients

^b^Percentage of total paediatric patients

^c^32 patients excluded from analysis of source of sepsis, due to incomplete history obtained. 5 patients judged to have more than one presumed source of sepsis

^d^4 patients deemed to have more than one presumed source of sepsis

^e^Other included: abdominal (n=2), gastrointestinal (n=1), trauma-related (n=1), head and neck (n=1)
