# Supplementary material for: Can the Xpert MRSA/SA BC assay be used as an antimicrobial stewardship tool? A prospective assay validation and descriptive impact assessment study in a South African setting
Source: BMC Infect Dis. 2021 Feb 15;21:177. doi: 10.1186/s12879-021-05857-7 (PMC7885373; doi:10.1186/s12879-021-05857-7)
Supplement: Supplementary file 3 — Additional file 3: Table S3. Patients with Gram positive cocci in clusters on blood culture and adequate antibiotic history who were excluded from impact analysis due to discordance between culture result, clinical history and antibiotic therapy (n = 3). Further details on the three patients with adequate clinical history pertaining to antibiotic use who were excluded from analysis due to inconsistency between culture result, clinical history and antibiotic therapy. [file 12879_2021_5857_MOESM3_ESM.docx]

**Additional file 3 (Supplementary material):**

Can the Xpert MRSA/SA BC assay be used as an antimicrobial stewardship tool? A prospective assay validation and descriptive impact assessment study in a South African setting

*Supplementary Table 3: Patients with Gram positive cocci in clusters on blood culture and adequate antibiotic history who were excluded from impact analysis due to discordance between culture result, clinical history and antibiotic therapy (n=3)*

|  | Blood culture result | Antibiotic therapy | Reported suspected source of sepsis |
| --- | --- | --- | --- |
| Patient 1 | MSSA | Empiric ceftriaxone, changed to vancomycin on final culture result | Unknown |
| Patient 2 | CoNS | Empiric vancomycin | Nosocomial pneumonia |
| Patient 3 | CoNS | Empiric ceftriaxone, changed to clindamycin on final culture result | Urinary tract infection |

MSSA: methicillin-sensitive *S. aureus*, CoNS: coagulase-negative staphylococci
