# Supplementary material for: Can the Xpert MRSA/SA BC assay be used as an antimicrobial stewardship tool? A prospective assay validation and descriptive impact assessment study in a South African setting
Source: BMC Infect Dis. 2021 Feb 15;21:177. doi: 10.1186/s12879-021-05857-7 (PMC7885373; doi:10.1186/s12879-021-05857-7)
Supplement: Supplementary file 4 — Additional file 4: Table S4. Evolution of antimicrobial therapy on Gram stain and culture result availability, in patients with Gram positive cocci in clusters on blood culture and known antibiotic history (n = 178). Action (modification/de-escalation/no change) in the 178 patients with known antibiotic history, in response to Gram stain and culture result release. [file 12879_2021_5857_MOESM4_ESM.docx]

**Additional file 4 (Supplementary material):**

Can the Xpert MRSA/SA BC assay be used as an antimicrobial stewardship tool? A prospective assay validation and descriptive impact assessment study in a South African setting

*Supplementary Table 4: Evolution of antimicrobial therapy on Gram stain and culture result availability, in patients with Gram positive cocci in clusters on blood culture and known antibiotic history (n=178)*

|  | MRSA (n=11) | | MSSA (n=34) | | CoNS (n=133) | |
| --- | --- | --- | --- | --- | --- | --- |
|  | **In response to Gram stain result** | **In response to final culture result** | **In response to Gram stain result** | **In response to final culture result** | **In response to Gram stain result** | **In response to final culture result** |
| Modification^a^ | 2 | 6 | 2 | 7^c^ | 8 | 1 |
| De-escalation^b^ | 0 | 7 | 0 | 18 | 0 | 13 |
| No change | 9 | 3 | 32 | 9 | 125 | 119 |

MRSA: methicillin-resistant *S. aureus*; MSSA: methicillin-susceptible *S. aureus*; CoNS: coagulase-negative staphylococci

^a^Modification classified as a change from an ineffective to a more effective agent, or the addition of a semisynthetic penicillin (methicillin susceptible *S. aureus*) or glycopeptide (methicillin-resistant *S. aureus* or CoNS)

^b^De-escalation classified as a change in antibiotic to a narrower-spectrum, targeted antistaphylococcal agent, or cessation of some or all antibiotics

^c^Of the 7 patients who had a modification of antibiotic therapy on the basis of the final culture result, 3 were changed from ceftriaxone to cefepime. These were classified as modification as cefepime was judged to be more effective anti-MSSA cover in the context of the clinical history; however, ceftriaxone was still regarded as empirically having anti-MSSA activity
