# Supplementary material for: Can the Xpert MRSA/SA BC assay be used as an antimicrobial stewardship tool? A prospective assay validation and descriptive impact assessment study in a South African setting
Source: BMC Infect Dis. 2021 Feb 15;21:177. doi: 10.1186/s12879-021-05857-7 (PMC7885373; doi:10.1186/s12879-021-05857-7)
Supplement: Supplementary file 5 — Additional file 5: Table S5. Contribution of antibiotic agents to the days of antibiotic therapy saved in patients with known antibiotic history (n = 178). A summary of antibiotic days potentially saved, by antibiotic type, in this cohort with use of the Xpert MRSA/SA BC assay. [file 12879_2021_5857_MOESM5_ESM.docx]

**Additional file 5 (Supplementary material):**

Can the Xpert MRSA/SA BC assay be used as an antimicrobial stewardship tool? A prospective assay validation and descriptive impact assessment study in a South African setting

*Supplementary Table 5: Contribution of antibiotic agents to the days of antibiotic therapy saved in patients with known antibiotic history (n=178)*

| Agent | Days saved | % | 95% confidence interval |
| --- | --- | --- | --- |
| Third-generation cephalosporins | 20 | 37.0% | 24.6-51.3% |
| Carbapenems | 8 | 14.8% | 7.1-27.7% |
| Aminoglycosides^a^ | 7 | 13.0% | 5.8-25.5% |
| Glycopeptides | 5 | 9.3 % | 3.5-21.1% |
| Beta-lactam beta-lactamase inhibitor combinations | 4 | 7.4% | 2.4-18.7% |
| Aminopenicillin | 4 | 7.4% | 2.4-18.7% |
| Macrolide | 3 | 5.6% | 1.5-16.3% |
| Semisynthetic penicillin | 1 | 1.9% | 0.1-11.2% |
| Fluoroquinolone | 1 | 1.9% | 0.1-11.2% |
| Trimethoprim-sulphamethoxazole | 1 | 1.9% | 0.1-11.2% |
| Total | **54** | **100%** | **-** |

^a^Aminoglycosides administered as part of combination therapy in all patients
